# Enhancing African coelacanth monitoring using environmental DNA

**DOI:** 10.1098/rsbl.2024.0415

**Published:** 2024-10-23

**Authors:** Jody-Carynn Oliver, Peter Shum, Stefano Mariani, Kerry Jennifer Sink, Ryan Palmer, Gwynneth Felicity Matcher

**Affiliations:** ^1^ Department of Ichthyology and Fisheries Science, Rhodes University, Makhanda, South Africa; ^2^ South African Institute for Aquatic Biodiversity (SAIAB), Makhanda, South Africa; ^3^ School of Biological and Environmental Sciences, Liverpool John Moores University, Liverpool L3 3AF, UK; ^4^ South African National Biodiversity Institute (SANBI), Cape Town, South Africa; ^5^ Institute for Coastal and Marine Research, Nelson Mandela University, Gqeberha (Port Elizabeth), South Africa; ^6^ Department of Biochemistry and Microbiology, Rhodes University, Makhanda, South Africa

**Keywords:** eDNA, coelacanth, marine biodiversity, DNA monitoring, management, South Africa

## Abstract

Coelacanths are rare, elusive, ancient lobe-finned fish species, residing in poorly accessible tropical marine caves and requiring close monitoring and protection. Environmental DNA (eDNA) approaches are being increasingly applied in the detection of rare and threatened species. Here we devise an eDNA approach to detect the presence of African coelacanths (*Latimeria chalumnae*) off the eastern coast of South Africa. Novel coelacanth-specific primers were designed to avoid cross-amplification with other fish lineages and validated for specificity. These primers were tested on field samples in conjunction with remotely operated vehicle (ROV) visual surveys. Samples were collected from a known coelacanth habitat and two adjacent slope habitats a few kilometres apart. Coelacanth DNA was detected from three of 15 samples collected. Two of these positive eDNA detections occurred in the presence of coelacanths, as evidenced by ROV footage, while the third positive detection was at a station where coelacanths had not been previously observed. eDNA detections are discussed in relation to the species’ metabolic rate, movement patterns and population size, as well as the local oceanographic features. We demonstrate that eDNA can provide a non-invasive method to extend the knowledge of coelacanth distribution ranges and boost research efforts around these iconic fishes.

## 1. Introduction

Living coelacanths are rare species of the genus *Latimeria*, the only extant genus of the ancient actinistian fishes, which, alongside six lungfish species, represent the living sister group of all tetrapods [1–3]. There are only two extant species: the African coelacanth, *Latimeria chalumnae* [4], inhabiting the Western Indian Ocean (WIO), and the Indonesian coelacanth, *Latimeria menadoensis* [5], distributed mostly between Sulawesi and Papua. Both known species occur in submarine caves along steep slopes in moderately deep water [6,7]. The African coelacanth is listed in Appendix I of CITES and rated as ‘critically endangered’ by the International Union for Conservation of Nature (IUCN) [8]. Increasing catches of coelacanths in shark gillnets in Tanzania and Madagascar are prompting calls for increased protection of this endangered species [9–11].

The first live coelacanth known to science was caught by a trawler fishing between depths of 40 and 70 m off the Chalumna River near eMonti, East London, South Africa, in 1938 [4]. In the following decades, more coelacanths were recorded further north from tropical waters in the Comoros [12], Mozambique [13], Madagascar [11,14] and Kenya [15]. A second species of coelacanth, *L. menadoensis*, was later discovered in the Indonesian archipelago [5,6]. It was only in October 2000 that a second South African coelacanth location was discovered, when divers spotted three individuals off Sodwana Bay [16], approximately 870 km north of the first known capture location. More recently, another coelacanth location off Umzumbe in southern KwaZulu-Natal [17] was found, approximately 480 km north and 430 km south of the eMonti and Sodwana locations, respectively. These findings suggest that coelacanths may be more widespread than originally thought and that the first specimen caught off East London in 1938 may not have been a stray as previously proposed [17]. Since 1938, this iconic species has become scientifically and culturally emblematic and a flagship for evolutionary thinking and conservation action [10,18]; therefore, it is vital to know the extent of their distribution in order to effectively secure their long-term future.

Traditional survey methods for monitoring rare and threatened species are not only expensive and time consuming but also tend to miss the more elusive taxa [19,20]. The use of environmental DNA (eDNA) methods presents an ideal solution, as this approach relies on the isolation of DNA shed by organisms into the surrounding environment [21], thereby providing a non-invasive, real-time snapshot of species presence and a relatively cost-effective and rapid method for assessing and monitoring biodiversity [22,23]. Targeted eDNA sampling may, therefore, prove to be an effective and versatile tool to expand the frequency and granularity with which coelacanth populations are mapped and monitored. Here, we design a tailored molecular assay for this iconic genus and validate the method using conventional polymerase chain reaction (PCR) on reference tissue samples, as well as by amplifying and sequencing eDNA from natural water collections from Sodwana Bay in the iSimangaliso marine protected area (MPA) in KwaZulu-Natal, South Africa.

## 2. Methods

### (a) Environmental DNA assay design and *in vitro* testing

The *L. chalumnae* species-specific primers were designed based on four mitogenomes retrieved from GenBank (nt). Primer3 [24] implemented in Geneious R11.0.3 (https://www.geneious.com/) was used to amplify approximately 194 base pair (bp) fragments anchored at the tRNA-Pro and spanning a section of the D-loop region of the mitochondrial DNA (mtDNA). The primer sequences are as follows: *Lati602F*: 5′-ACT TTT ACC CTT AGC TCC CAA AG-3′ and *Lati776R*: 5′-CGC CCT AAA TGT GGT GTG AC-3′; they were designed to perfectly bind to both *Latimeria* species and exclude all other major fish lineages and were first tested *in silico* using ecoPCR [25] against the EMBL R142 database.

For the *in vitro* testing of the primer set, gill samples of *L. chalumnae* collected from Tanga, Tanzania, in September 2003 (which had been frozen at −80°C in RNALater (Ambion)), were used to extract total genomic DNA (gDNA), using the E.Z.N.A.^®^ Tissue DNA Kit (Omega Bio-tek). DNA concentration of the extracted gDNA, measured using the NanoDrop™ 2000 (ThermoFisher Scientific™), was 71 ng µl^−1^ (260/280 ratio: 1.99). PCR amplifications were performed in a 15 µl reaction volume containing 71 ng of DNA, 7.5 µl of Taq DNA Polymerase 2× Master Mix RED (Ampliqon), 0.3 µl of each primer (10 µM) and 5.9 µl of molecular grade H_2_O. A gradient PCR was first performed to determine the optimal annealing temperature of the designed primers. The PCR cycling profile comprised an initial denaturation step at 98°C for 2 min, 30 cycles of denaturation (98°C for 1 min), annealing (52–57°C for 1 min) and extension (72°C for 1 min) and a final extension at 72°C for 7 min. PCR amplification success was checked by 1× Tris-borate-EDTA (TBE) gel electrophoresis in 1% agarose and stained with ethidium bromide (0.05 µg ml^−1^).

To test the specificity of the primer pair, an *in vitro* test was carried out (using the optimal thermocycling conditions identified through the experiment above, with an annealing temperature of 57°C) with DNA of seven species representing divergent fish groups that are common along the eastern South African coast, namely, common smooth-hound, *Mustelus mustelus* (Elasmobranchii: Carcharhiniformes); blue stingray, *Dasyatis chrysonota* and common eagle ray, *Myliobatis aquila* (Elasmobranchii: Myliobatiformes); undulated moray eel, *Gymnothorax undulatus* (Teleostei: Anguilliformes); black musselcracker, *Cymatoceps nasutus* and 74 seabream, *Polysteganus undulosus* (Teleostei: Acanthuriformes); and evileye pufferfish, *Amblyrhynchotes honckenii* (Teleostei: Tetraodontiformes). In addition, the sensitivity of the specific marker was determined *in vitro* with serial dilutions from 1 to 10 000 000 of *L. chalumnae* DNA from a known concentration (70 ng µl^−1^). The specificity test was repeated using the high-fidelity DNA polymerase, KAPA HiFi HotStart ReadyMix (2X; KAPA Biosystems).

### (b) Study area for field validation

**Figure 1.**
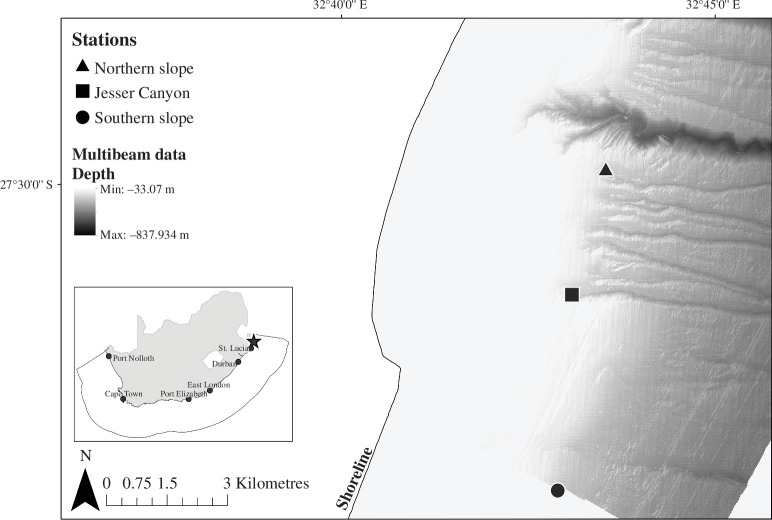
Sampling locations of environmental water samples collected offshore of Sodwana Bay within iSimangaliso MPA. Refer to table 1 for more information on sampling stations. Multibeam bathymetry data from Ramsay & Miller [26].

This study focused on one of the known South African coelacanth locations, Jesser Canyon and its adjacent slope, an area off Sodwana Bay within the iSimangaliso MPA, northern KwaZulu-Natal, South Africa [7]. Sampling occurred from April to May 2022 on the NRF-SAIAB research vessel Phakisa. Three sampling locations were visited: Jesser Canyon as a known coelacanth location with an average depth of 111 m, a slope habitat approximately 3 km north of the canyon with an average depth of 153 m (Northern slope) and a slope habitat 5 km south of the canyon with an average depth of 93.2 m (Southern slope; figure 1). Sampling in deep submarine caves below 100 m depth requires specialized and expensive equipment, technical expertise and access to offshore research vessels. Jesser Canyon is a submarine canyon with four known coelacanth caves and individual coelacanths have been recorded moving between this canyon and Wright Canyon [7], a canyon with six known coelacanth locations 4 km north of Jesser Canyon. The northern slope station is steep and has rocky and sandy seabed. The southern slope station is sandy, less steep and part of an oceanographic monitoring array.

**Table 1.** Overview of eDNA sampling stations in the iSimangaliso MPA, with each station being classified as known or unknown coelacanth habitat based on previous surveys. For each station, the table indicates whether coelacanth eDNA was detected, and for the stations where simultaneous visual surveys were conducted, the number of observed coelacanths is given.

station	location name	collection date	station no.	habitat	coelacanth habitat (known/unknown)	eDNA detection	no. coelacanth observed concurrently by ROV
1	slope north of Jesser Canyon	28 Apr 2022	DCN_CTD1	deep slope	unknown	no	no visual assessment^a^
		28 Apr 2022	DCN_CTD1	deep slope	unknown	no	no visual assessment^a^
2	Jesser Canyon	29 Apr 2022	DCN009	cave in canyon (cave a)	known	yes	4
		29 Apr 2022	DCN010	cave in canyon (cave a)	known	yes	4
		2 May 2022	DCN014	cave in canyon (cave b)	known	no	0
		2 May 2022	DCN015	cave in canyon (cave b)	known	no	0
		2 May 2022	DCN016	cave in canyon (cave b)	known	no	1
		2 May 2022	DCN017	cave in canyon (cave a)	known	no	1
		2 May 2022	DCN018	cave in canyon (cave c)	known	no	0
		2 May 2022	DCN_CTD2	in canyon head	known	no^a^	no visual assessment^a^
		2 May 2022	DCN_CTD2	in canyon head	known	no^a^	no visual assessment^a^
		2 May 2022	DCN_CTD2	in canyon head	known	no	no visual assessment^a^
3	slope south of Jesser Canyon	5 May 2022	DCN_CTD5	deep slope	unknown	no^a^	no visual assessment^a^
		5 May 2022	DCN_CTD5	deep slope	unknown	no^a^	no visual assessment^a^
		5 May 2022	DCN_CTD5	deep slope	unknown	yes^a^	no visual assessment^a^

^a^ Environmental sample was collected by conductivity–temperature–depth with a rosette, and thus, there is no visual footage present during the time of collection for visual confirmation of coelacanths.

### (c) Water collection, filtration and DNA extraction

**Figure 2.**
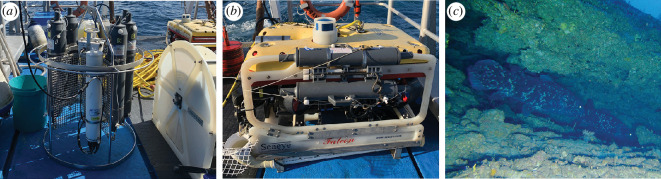
Seawater sampling was done using (*a*) a CTD instrument with a rosette containing six 4 l Niskin bottles, which was applied in the canyon head and along the deep slopes, and (*b*) the ROV with two 1.5 l Niskin bottles attached to the frame, which was applied inside Jesser Canyon. The ROV was also used to capture the image in (*c*) of the African coelacanths inside one of the sampled caves. Photographic credits: (*a*) and (*b*) Jody-Carynn Oliver and (*c*) ACEP Deep Connections.

Seawater for the analysis of eDNA was collected using a remotely operated vehicle (ROV) of the model SAAB SeaEye Falcon and a conductivity–temperature–depth (CTD) instrument of the model Sea-Bird SBE 19plusV2, each with affixed Niskin bottles (figure 2*a*,*b*). Due to logistical and financial constraints, the CTD was used to collect water in the areas where ROV surveys were not prioritized. This study was one part of a broader multidisciplinary research project covering other disciplines, communities and species, and ‘non-cave’ water samples were opportunistically obtained at stations subject to oceanography data collection using CTD, which also provided a larger volume of water. In the caves, seawater was collected both in close proximity to observed coelacanths (figure 2*c*) as well as when coelacanths were absent (table 1); water was also collected from unknown but potential coelacanth habitats. A total of 15 eDNA samples, with four negative field controls, were collected. At the sampling stations, 3 l (ROV collected) and/or 5 l (CTD collected) of seawater were filtered (see table 1) through 0.22 μm Sterivex filters (Millipore) using a vacuum pump on board the research vessel Phakisa. Filters were then capped and placed in sterile ziplock bags and stored in a −20°C freezer on board the vessel.

DNA extractions were performed in a sterile eDNA laboratory, using the E.Z.N.A. Tissue DNA Kit (Omega Bio-tek) following the manufacturer’s protocol. The DNA quantities were assessed using ThermoFisher Scientific NanoDrop technology. Pre- and post-PCR steps were conducted in separate laboratories.

### (d) Contamination control protocol

To monitor potential contamination, controls were used throughout all stages of the study. All water collection bottles and filtering equipment were sterilized prior to and after use, using a 10% bleach solution. Negative controls consisting of 1 l reverse osmosis water were simultaneously filtered with field samples on board the research vessel. All subsequent preparation of metabarcoding amplicon libraries for sequence analysis was carried out by processing only samples from one station at a time, followed by sterilization of the laboratory. All laboratory surfaces and equipment were sterilized with 10% bleach solution followed by 30 min under ultraviolet light. Pre- and post-PCR processes were conducted in physically isolated laboratories, where no coelacanth tissue had ever been present. Negative controls included field sample controls (which also served as extraction controls) and PCR controls (i.e. sterile water in place of extracted template DNA). All negative controls were processed in an identical manner to that of the test samples and sequenced alongside test samples. Furthermore, to prevent false positives, no positive control DNA templates were included in any PCRs, and *in vitro* testing of the primer set was conducted in a separate building (at the South African Institute for Aquatic Biodiversity (SAIAB; see [27])).

### (e) Validation of the *Latimeria chalumnae* environmental DNA assay

PCR amplifications using the *Lati602F* and *Lati776R* primers were performed in a 25 µl reaction volume containing 5 µl of DNA, 12.5 µl of KAPA HiFi HotStart ReadyMix (2X; KAPA Biosystems), 0.5 µl of each primer (10 µM) and 6.5 µl of molecular grade H_2_O. For environmental samples, the PCR cycling profile comprised two steps, with the second step applied to introduce Illumina adaptors to allow for multiplexing during subsequent sequence analysis. The first PCR cycle set had an initial denaturation step at 98°C for 5 min, 45 cycles of denaturation (98°C for 30 s), annealing (64°C for 1 min) and extension (72°C for 45 s) and a final extension at 72°C for 5 min. The second PCR cycle set had an initial denaturation step at 98°C for 5 min, 10 cycles of denaturation (98°C for 30 s), annealing (64°C for 30 s) and extension (72°C for 30 s) and a final extension at 72°C for 5 min. Three technical PCR replicates were performed for each of the 22 analysed samples (consisting of 15 biological replicates and seven negative controls). The success of PCR amplifications was assessed by gel electrophoresis in 1% agarose. To validate that the target band visualized by agarose gel electrophoresis was indeed coelacanth DNA and to check for cross-species detection, all PCR products, including negative controls, were sequenced. Technical replicate PCR products were pooled and purified with Mag-Bind^®^ TotalPure NGS bead (2X bead : sample ratio), following the manufacturer’s protocol (Omega Bio-tek). Indexing of samples was carried out using the v2 Nextera XT indices (Illumina), followed by purification using Mag-Bind TotalPure NGS magnetic beads. The resultant purified amplicon libraries were quantified using the Qubit dsDNA High Sensitivity Assay kit (Invitrogen) on a Qubit 3.0 fluorometer (Life Technologies). Amplicon libraries were then pooled in an equimolar manner to a final concentration of 4 nM. The library was quantified by quantitative PCR (qPCR) using the NEBNext Library Quant Kit for Illumina (New England Biolabs) to determine the efficiency of the addition of index adaptors and to ensure the accurate quantification of the pooled library. A final library of 10 pM with 15% PhiX spike-in was sequenced using the MiSeq V2, 500-cycle reagent kit (Illumina) on the Illumina MiSeq platform at the Aquatic Genomics Research Platform at the SAIAB.

### (f) Bioinformatic processing of sequencing data

Mothur v. 1.48.0 [28] was used for quality checking and filtering of sequence data (Sequence Read Archive accession numbers: SRX25369892-SRX25369912 and SRX25989550). Primers, short reads, reads containing any ambiguous nucleotides and chimeras (identified using vsearch [29]) were removed from the dataset. For the taxonomic assignment of the curated dataset, the NCBI’s Standalone BLAST software was used [30]. A blastn query was conducted against a reference database containing all eight available D-loop sequences of *L. chalumnae* downloaded from GenBank (accession numbers: NW_005819070.1, NW_005819123.1, NW_005819306.1, AB257296.1, AB257297, NC_001804.1, Z21921.1 and M87534.1). Samples that gave positive *L. chalumnae* detection were then subjected to a BLAST search against the entire NCBI nr/nt database (v. 2.11) to serve as a verification step for the accuracy and specificity of the initial results.

## 3. Results

**Figure 3.**
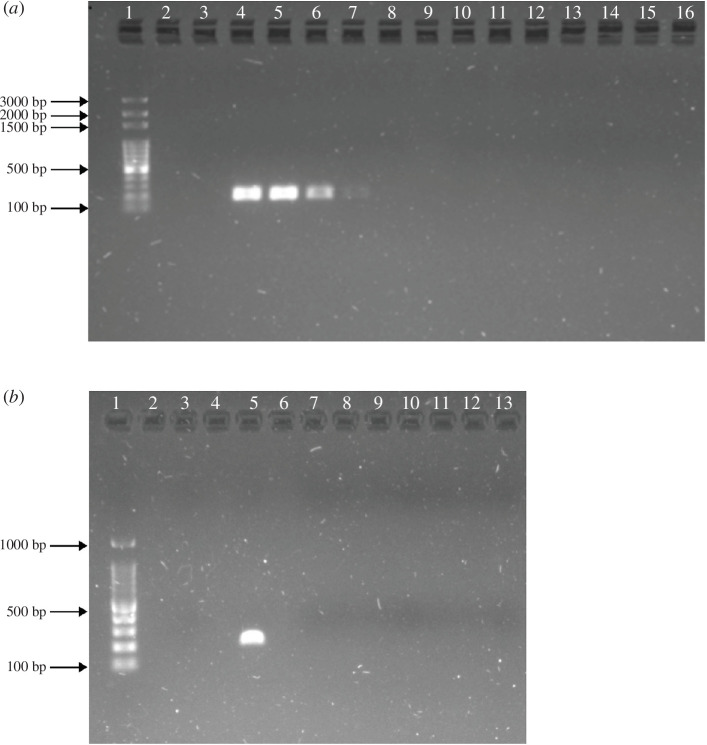
*In vitro* testing of the coelacanth primer assay visualized on a 1% agarose gel using the Solis 100 bp ladder (lane 1). (*a*) Primer sensitivity of dilution 1 : 10 000, containing coelacanth DNA (71 ng µl^−1^; lanes 4–8) and specificity test across five major fish lineages: lane 10: *Mu. mustelus* (Selachimorpha), lane 11 and lane 12: *D. chrysonota* and *My. aquila* (Batoidea); lane 13: *G. undulatus* (Elopomorpha); and two families within the megadiverse Percomorpha: lane 14 and lane 15: *C. nasutus* and *P. undulosus* (Sparidae) and lane 16: *A. honckenii* (Tetraodontidae). Lane 3 is a negative control, with Ampliqon DNA polymerase. (*b*) Specificity test across the same seven fish species using a high-fidelity polymerase, KAPA HiFi HotStart ReadyMix (2X; lanes 7–13: order of species is the same above, lane 3: negative control and lane 5: positive control).

The detection threshold of this assay based on PCR visualization of amplified coelacanth DNA in agarose gel was 0.007 ng µl^−1^, since a weak but visible band was detected in the dilution 1 : 10 000 from a sample with a concentration of 71 ng µl^−1^ (figure 3*a*). The eDNA assay proved to be species-specific as it did not cross-amplify any of the known major fish groups with either of the two DNA polymerases (figure 3*b*).

All field and PCR controls were clear of contamination. Coelacanth eDNA was detected in three of the 15 water samples collected, including two samples from known coelacanth locations and one site south of Jesser Canyon. High-throughput sequencing generated 346 322 reads out of these 15 water samples; after curation and quality control, 62 092 remained, of which 33.2% (20 644 reads) could be taxonomically assigned to *L. chalumnae* at greater than 99% sequence identity, with no assignments to any other fish or vertebrate species (see electronic supplementary material, tables S1 and S2).

The two positive detections of coelacanth eDNA at the known coelacanth locations (DCN009: 18 495 reads; DCN010: 20 reads) were from water samples collected by the ROV, while four coelacanths were present at the time of collection (table 1). In instances where only one coelacanth was observed to be present by the ROV, no coelacanth eDNA was detected (table 1). The third detection was from a water sample collected by CTD with a rosette at the station 5 km south of Jesser Canyon (DCN_CTD5: 2129 reads; table 1; figure 1).

## 4. Discussion

The detection of coelacanth eDNA from open-water samples demonstrates the power of eDNA approaches to reveal the presence of this rare and endangered species in new locations. This is consistent with a rapidly growing body of research that highlights the value of eDNA-based species biomonitoring in marine systems [31]. The demonstration of the utility of this approach in the field suggests that it is likely to play a significant role in improving our understanding of coelacanth distributions, with implications for coelacanth research and conservation strategies.

The developed assay is efficient and coelacanth-specific, as per *in vitro* testing on tissues and validation with environmental samples (figure 3). This is further validated by the Illumina sequencing of positive detections where no extra sequences of other vertebrate species were detected, corroborating the specificity of this assay. Based on *in silico* analysis, this assay is also expected to amplify the DNA of the Indonesian coelacanth, *L. menadoensis*, making it a useful non-invasive tool for future research and management of both extant coelacanth species.

While the detection of coelacanth eDNA sequences near the cave where four coelacanths were sighted is not surprising, the signal detected at the station 5 km south of Jesser Canyon was remarkable. Here, no simultaneous ROV observations were available to confirm the detection, but the absence of contamination in all negative controls indicates the true presence of coelacanth eDNA. Given that the water was collected along the same continental margin and considering coelacanth movement behaviour between Jesser and Wright Canyon to the north [7], as well as the persistence of eDNA in marine waters [23,32], it signifies either that coelacanths had recently been present at the time of sampling or that trace DNA molecules were transported from the known coelacanth location by the strong north-to-south Agulhas current. The acquisition of coelacanth DNA in water collected by the ROV indicates that the targeted water collection using Niskin bottles mounted on the ROV was an effective sampling method; however, there were also false-negative instances (table 1): two collection events where single coelacanths were observed by the ROV, but no coelacanth eDNA was detected. This may be due to the low number of coelacanths present at the time of collection (i.e. single individuals) and/or a low eDNA shedding rate. The presence of false negatives is a common challenge with eDNA studies [33–36], which indicates that future research efforts could improve this assay by tailoring it to qPCR or droplet digital PCR (ddPCR) protocols, which are typically more precise, with higher degrees of sensitivity [37–40].

The current extent of coelacanth distribution in the WIO covers a considerable area, as shown by the recent coelacanth sightings 430 km south of the South African breeding population in iSimangaliso MPA [17]. Despite intensive research on this iconic species, studying their distribution is particularly challenging. This eDNA assay may assist future targeted coelacanth surveys—especially in the typical cases where the use of ROV-equipped vessels is too expensive—to further understand coelacanth distributions in the WIO. In the near future, eDNA may establish itself as a versatile and cost-effective tool to assist other African countries, such as Mozambique [13] and Kenya [15], in determining whether the single specimens caught off their coasts are part of unknown native populations, for example, eDNA sampling in steep marine areas around Madagascar [11,41] and islands such as Pemba, Europa, the Gloriosa Islands and Bassas da India could offer new insights into coelacanth distribution, dispersal and ecology. The eDNA approach may just offer the flexibility and scalability—especially if associated with participatory or citizen science activities—that will enable more frequent and extensive mapping and monitoring initiatives aimed at effective management action. We also hope to see this marker tested on water samples around the Indonesian archipelago in the near future.

## Data Availability

Raw sequence data and metadata are available on the NCBI Sequence Read Archive (SRA) under BioProject PRJNA1137142. The scripts used in this study for bioinformatic processing are available on Zenodo [42]. Supplementary material is available online [43].
